# Prediction models for hormone receptor status in female breast cancer do not extend to males: further evidence of sex-based disparity in breast cancer

**DOI:** 10.1038/s41523-023-00599-y

**Published:** 2023-11-08

**Authors:** Subarnarekha Chatterji, Jan Moritz Niehues, Marko van Treeck, Chiara Maria Lavinia Loeffler, Oliver Lester Saldanha, Gregory Patrick Veldhuizen, Didem Cifci, Zunamys Itzell Carrero, Rasha Abu-Eid, Valerie Speirs, Jakob Nikolas Kather

**Affiliations:** 1 Institute of Medical Sciences, University of Aberdeen, Aberdeen, UK; 2 Aberdeen Cancer Centre, University of Aberdeen, Aberdeen, UK; 3 Else Kröner Fresenius Centre for Digital Health, Carl Gustav Carus Faculty of Medicine, Technical University of Dresden, Dresden, Germany; 4 Department of Medicine III, University Hospital RWTH (Rheinisch-Westfälische Technische Hochschule) Aachen, Aachen, Germany; 5 Department of Medicine I, University Hospital and Faculty of Medicine, Technical University of Dresden, Dresden, Germany; 6 Institute of Dentistry, University of Aberdeen, Aberdeen, UK; 7 Division of Pathology and Data Analytics, Leeds Institute of Medical Research at St. James’s, University of Leeds, Leeds, UK

**Keywords:** Breast cancer, Prognostic markers

## Abstract

Breast cancer prognosis and management for both men and women are reliant upon estrogen receptor alpha (ERα) and progesterone receptor (PR) expression to inform therapy. Previous studies have shown that there are sex-specific binding characteristics of ERα and PR in breast cancer and, counterintuitively, ERα expression is more common in male than female breast cancer. We hypothesized that these differences could have morphological manifestations that are undetectable to human observers but could be elucidated computationally. To investigate this, we trained attention-based multiple instance learning prediction models for ERα and PR using H&E-stained images of female breast cancer from the Cancer Genome Atlas (TCGA) (*n* = 1085) and deployed them on external female (*n* = 192) and male breast cancer images (*n* = 245). Both targets were predicted in the internal (AUROC for ERα prediction: 0.86 ± 0.02, *p* < 0.001; AUROC for PR prediction = 0.76 ± 0.03, *p* < 0.001) and external female cohorts (AUROC for ERα prediction: 0.78 ± 0.03, *p* < 0.001; AUROC for PR prediction = 0.80 ± 0.04, *p* < 0.001) but not the male cohort (AUROC for ERα prediction: 0.66 ± 0.14, *p* = 0.43; AUROC for PR prediction = 0.63 ± 0.04, *p* = 0.05). This suggests that subtle morphological differences invisible upon visual inspection may exist between the sexes, supporting previous immunohistochemical, genomic, and transcriptomic analyses.

## Introduction

Male breast cancer (MBC) is a rare condition that accounts for approximately 1% of all breast cancer cases worldwide^1,2^. Its clinical management generally follows established strategies evidenced from female breast cancer (FBC). However, this may not be an optimal approach, as mounting evidence shows sex-specific differences in the molecular make-up, prognostic factors, and clinical demographics in BC^3–5^.

For both sexes, prognostication and treatment decision making is dependent upon the expression profiles of the nuclear hormone receptors estrogen receptor alpha (ERα) and progesterone receptor (PR), currently determined by immunohistochemistry. High expression of ERα and PR are both predictors of improved outcome in MBC, associated with improved overall and disease-free survival, older age of diagnosis, low mitotic index, and lower pathological stage^6–13^. The expression of these receptors is notably different between MBC and FBC. Contrary to females, BC in males is almost universally ERα positive (95% in MBC vs. 75% in FBC). PR positivity is observed in 82% of MBC and 65% of FBC cases^1,5,6,14,15^.

Chromatin binding characteristics of ERα and PR differ by sex. In MBC, PR binding sites often lack ERα, while in females, PR can modulate ERα binding^16^. Adding to this evidence, a hierarchical clustering study using immunohistochemical data showed separate clusters of ERα and PR independent of each other in MBC. However, the opposite was observed in FBC, where ERα and PR profiles clustered together^17^. Additionally, mathematical modelling of immunohistochemical staining has failed to show any continuous dependence effect of PR on ERα for MBC, in direct contradiction to FBC^18^.

Although sex-specific molecular differences in breast cancer have been demonstrated in multiple studies, there are no obvious morphological differences between MBC and FBC following visual inspection of haematoxylin and eosin (H&E) stained BC tissue sections. Consequently, MBC is classified and reported in the same way as FBC^1,19^, despite evidence that the well-known molecular subtypes in FBC may not be reflected in males. Such a non-specific approach is discordant with the differences in the distribution of histological subtypes. This also calls into question the existence of morphological disparities that manifest due to the sex-specific regulatory nature of BC which are not obvious to a human observer but may be elucidated using deep learning (DL) methods.

H&E-stained tissue sections are the primary diagnostic tool for cancer patients with solid tumours, and are commonly available and accessed with relative ease^20,21^. Recent work has shown that digitally scanned whole slide images (WSIs) of H&E-stained slides contain a wealth of previously hidden information which are not obvious to a human observer, but may be elucidated using computational models and could be of prognostic value^22–24^. The development of such artificial intelligence (AI) based algorithms means it is now possible to extract and quantify this information^23–25^.

Convolutional neural networks (CNNs) have been able to predict a range of clinical characteristics in FBC, such as grade, histological subtype, PAM50 intrinsic subtype, and ERα status^26–28^ directly from H&E-stained WSIs. Historically, biomarker prediction in computational pathology has employed the training of DL networks from pathological tumor annotated regions on the whole slide images. Only this region-of-interest (ROI) is then tiled, with each tile retaining the “tumor” annotation. Thus, “healthy” tissue or background is excluded from the analysis. However, this method may not be optimal due to two reasons. First, the ROI may contain regions that are not morphologically important for the target prediction^20,29–31^. Second, the tissue architectures surrounding the ROI that get rejected as background tiles may contain essential information for improved performance of the prediction model. To address these issues, our study employed a weakly supervised learning pipeline using slide-level annotations of biomarker status, which consider all types of tissue architectures in the WSI without any information loss and can be accessed with relative ease from patient records. For tile-to-slide level aggregation, we used a multiple instance learning pipeline with an attention component (attMIL)^32^.

In view of the evident sex-based differences of BC, we evaluated the efficacy of attMIL pipelines in predicting ERα and PR status in both MBC and FBC patients aiming to provide evidence of possible morphological differences between the sexes. We hypothesized that sex-based molecular differences may manifest in the morphological features contained in the tissue architecture which could be predictive of the hormone receptor status of the tumor in H&E-stained slides.

## Results

### attMIL models can predict ERα and PR status from H&E WSIs in FBC

We investigated whether attMIL-based DL models can predict hormone receptor status for ERα and PR in FBC WSIs. To do this, we used patient-level training and 5-fold cross-validation on the TCGA-BRCA FBC cohort (*n* = 1085) with and without colour normalisation. With normalisation, our predictions for ERα and PR showed mean area under the receiver operating characteristics (AUROCs) > 0.6 (0.86 ± 0.02, *p* < 0.001 and 0.76 ± 0.03, *p* < 0.001), respectively. Without normalisation, the respective AUROCs obtained were very similar: 0.86 ± 0.05 (*p* < 0.001) and 0.78 ± 0.02 (*p* < 0.001).

Next, we tested the hormone receptor prediction models on FBC WSIs independently from the training set by deploying them on a validation cohort of 192 FBCs. Performance of the models was assessed by the detection ability of both ERα and PR. With normalisation, the AUROCs were 0.78 ± 0.03 (*p* < 0.001) for ERα and 0.80 ± 0.04 (*p* < 0.001) for PR. Very similar AUROCs were returned without normalisation, which were 0.78 ± 0.05 (*p* < 0.001) and 0.76 ± 0.03 (*p* < 0.001) for ERα and PR, respectively.

**Fig. 1:**
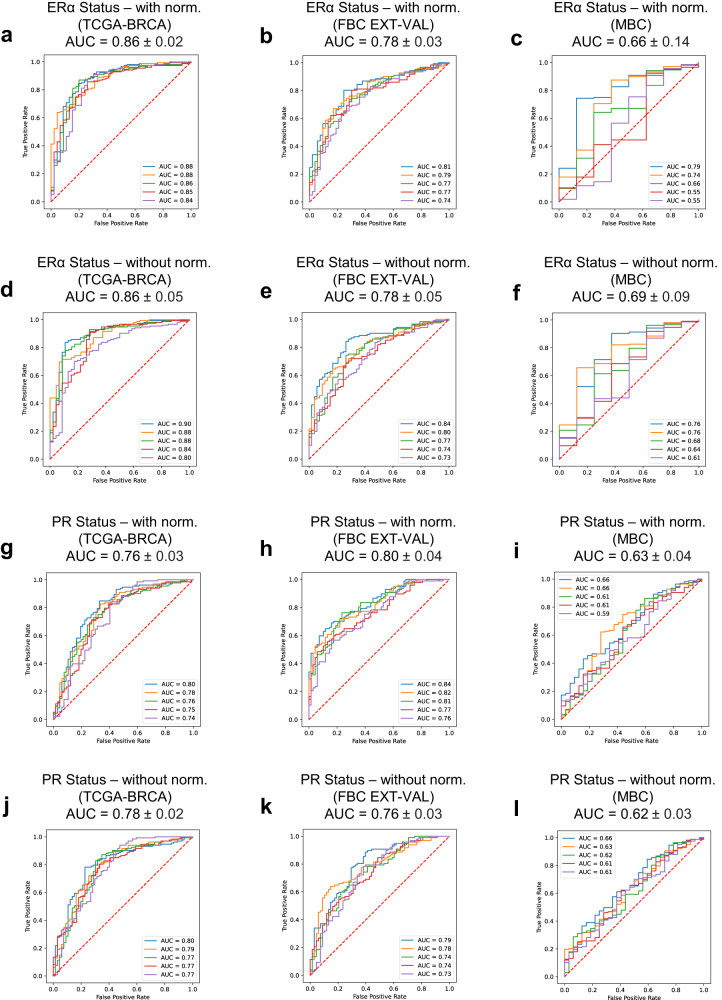
AUROCs of prediction models for ERα and PR. Biomarker prediction models in Female Breast Cancer (FBC) internal (TCGA-BRCA), external validation cohorts, and MBC cohort for ERα prediction model (**a**–**c**) with and (**d**–**f**) without normalisation; PR prediction model (**g**–**i**) with and (**j**–**l**) without normalisation. AUROCs indicate a model’s discriminatory power as follows: 0.5 = no discrimination; >0.5 to ≤0.7 = poor; >0.7 to ≤0.8 = acceptable; >0.8 to ≤0.9 = excellent; >0.9 = outstanding.

Collectively, these data show that attMIL-based prediction models for ERα and PR status in FBC can be predicted directly from H&E-stained WSIs. AUROCs for FBC cohorts are shown in Fig. 1a, b, d, e, g, h, j, k. Full accuracy metrics are provided in Supplementary Table 1.

### Prediction models trained on FBC images do not generalize to MBC

To test whether the attMIL-based prediction models are sex-invariant, we deployed the previously trained DL models on a combined set of MBC cases from 7 different centres (*n* = 198). For both ERα and PR, large performance drops were observed both with and without colour normalisation. With normalisation, AUROCs of 0.66 ± 0.14 (*p* = 0.43) and 0.63 ± 0.04 (*p* = 0.05) were returned, respectively. Without normalisation, AUROCs returned were very similar: 0.69 ± 0.09 (*p* = 0.08) and 0.62 ± 0.03 (*p* < 0.05) for ERα and PR, respectively. This indicated that the discriminatory power of prediction models for both ERα and PR trained on FBC images were poor when applied to males. ROCs for the MBC cohort are shown in Fig. 1c, f, i, l. Accuracy metrics are provided in Supplementary Table 1.

We qualitatively explored whether there were any constituent cohorts with especially low performance for either marker driving the overall underperformance of the prediction models in the combined MBC cohort. This was done by creating density plots for each target model for each cohort to see whether the distribution of the prediction scores was similar between the cohorts. The ERα prediction scores for the TCGA and PR prediction scores for BCNTB cases exhibited left-skewed distributions, indicating that the majority of the cases were accurately classified as ERα positive (for TCGA) and PR positive (for BCNTB) with high confidence. The prediction score distributions in the remaining cohorts were similar, and there was no evident skewing in any of the cohorts that could explain the overall subpar performance of the ERα and PR prediction models in the combined MBC cohort. Distribution of prediction scores in each MBC cohort have been shown in Supplementary Fig. 1.

### Hormone receptor prediction models in FBC are sensitive to the target they were trained to detect

**Fig. 2:**
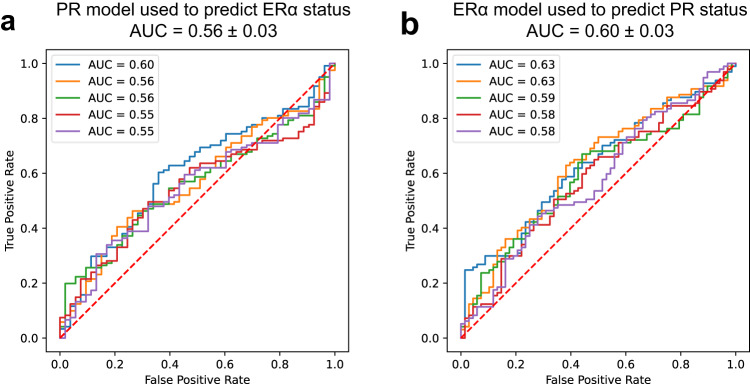
AUROCs of experiments designed to test sensitivity of each prediction model on different nuclear hormone receptors. AUROCs achieved on deploying (**a**) PR prediction model to detect ERα and (**b**) ERα prediction model to detect PR. AUROCs indicate a model’s discriminatory power as follows: 0.5 = no discrimination; >0.5 to ≤0.7 = poor; >0.7 to ≤0.8 = acceptable; >0.8 to ≤0.9 = excellent; >0.9 = outstanding.

We evaluated the sensitivity of DL-based prediction models to the biomarker target they were trained to detect by applying an ERα prediction model to detect PR status and vice versa on the external validation dataset of FBC. The AUROC for the ERα model detecting PR status was 0.56 ± 0.03 (*p* = 0.45). For the PR model detecting ERα status, it was 0.60 ± 0.03 (*p* = 0.06). Neither model achieved statistical significance nor exceeded the 0.6 baseline AUROC, indicating poor discriminatory power for the target they were not trained to detect. Figure 2 shows the ROCs for both experiments.

### attMIL model predictions for ERα and PR positivity are validated by immunohistochemistry in FBC but not in MBC

To better understand how the attMIL-based prediction models make decisions, we investigated the spatial distribution of prediction and attention scores. For ERα and PR positive cases, we also explored whether these distributions aligned with immunohistochemistry, in both FBC and MBC WSIs. These scores for ERα and PR were visualized separately on matched immunohistochemistry (IHC) WSIs. We also examined the spatial distribution of prediction and attention scores for ERα and PR negative FBC and MBC cases as well.

**Fig. 3:**
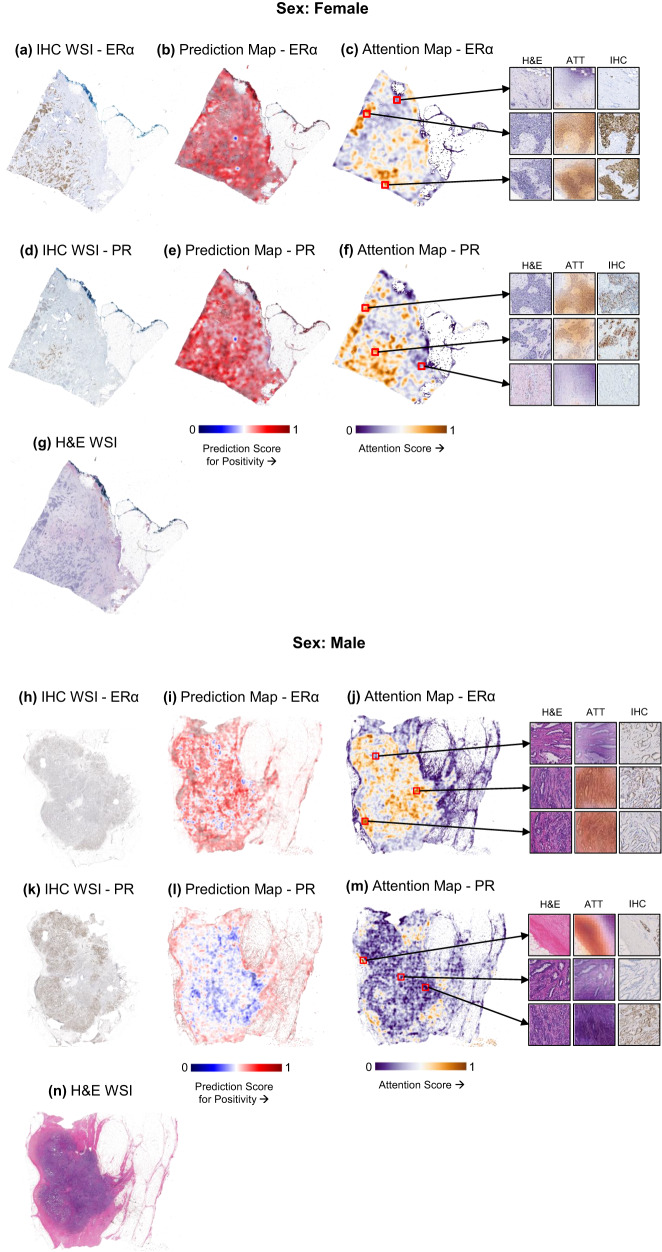
Heatmaps showing spatial resolution of attention and prediction scores in ERα and PR positive FBC and MBC WSIs, their concordance with corresponding IHC staining patterns, and the H&E WSIs from which these heatmaps were generated. FBC (top) and MBC (bottom) prediction models with respective adjacent views of (**a**, **h**) ERα IHC WSIs, (**b**, **i**) prediction score maps for ERα, (**c**, **j**) attention score maps for ERα, (**d**, **k**) PR IHC WSIs, (**e**, **l**) prediction score maps for PR, (**f**, **m**) attention score maps for PR, and (**g**, **n**) the H&E-stained WSIs from which these score maps were generated, along with magnified views of representative tiles for high and low attention regions with their corresponding regions in the IHC and H&E-stained WSIs. The attention maps showcase the relevant morphological features with high attention regions in gold and low attention regions in purple, irrespective of the final prediction. The prediction maps highlight the relevance of each tile in making a prediction of the target receptor positivity represented in red, and negativity in blue. The statuses of both target receptors were predicted correctly in the FBC WSI, and the high attention regions were concordant with receptor positivity for both ERα and PR when matched with the IHC WSI. In the MBC WSI, the overall ERα status was predicted correctly although certain areas within the WSI were predicted to be positive. Furthermore, high attention regions had no clear concordance with the IHC staining pattern. The same observation was made in the PR attention score pattern as well, and the overall prediction made was also incorrect. The FBC WSIs shown in this image are serial sections of the following order: (1) PR IHC, (2) ERα IHC, and (3) H&E-stain. The order of the serial sections of the MBC WSIs is: (1) H&E-stain, (2) ERα IHC, and (PR) PR IHC.

In FBC, the spatial resolution of the prediction score heatmaps were not focused on any specific region of the WSIs, irrespective of hormone receptor status. They represented the probability of each constituent tile being classified as positive or negative, resulting in a diffuse colour map (red or blue). In MBC, however, certain regions in the WSIs were predicted to be of the incorrect class, even if the overall classification matched the ground truth. These overall observations were true for either positivity or negativity of both target markers. This was especially evident for the PR model where large areas of the WSIs were predicted to be of the incorrect class in both the positive and negative examples. Representative examples of prediction score maps for ERα/PR positive cases in FBC are shown in Fig. 3b, e for ERα and PR prediction, respectively. In MBC, similar examples are shown in Fig. 3i, l for ERα and PR prediction. Prediction score maps for FBCs negative for ERα and PR can be found in Supplementary Fig. 2b, d, respectively. In MBC, similar examples are shown in Supplementary Fig. 2g, i for ERα and PR prediction, respectively.

The heatmaps showing the distribution of attention scores were more specific to certain regions in each WSI. In ERα and PR positive FBC, high attention regions were concentrated on tumor tissue for both markers, although to a lesser extent for PR. Matched IHC WSIs showed that the attention score maps are concordant with the staining patterns, especially for ERα. For PR, the attention score distribution was more diffuse than in the ERα map, and the corresponding PR IHC staining revealed less positivity compared to ERα. In hormone receptor positive MBC, high attention scores for ERα were limited to the tumor tissue, while the surrounding stromal regions received low attention scores. Low attention scores were returned for some areas in the tumor region as well. However, unlike in FBC, a clear concordance was not seen between high attention scores and receptor positivity, as some low attention regions also had ERα positivity. For PR, the entire tumor region had low attention scores. No concordance was observed between attention score patterns and IHC staining for PR. In hormone receptor negative FBC, high attention scores for ERα negative regions were quite diffuse, while those in areas of PR negativity were sharper. The opposite pattern was observed in hormone receptor negative MBC. Representative examples of attention score maps for hormone receptor positive cases in FBC are shown in Fig. 3c, f for ERα and PR prediction, respectively. In MBC, similar examples are shown for ERα and PR prediction (Fig. 3j, m). Attention maps showing FBC ERα and PR negative cases can be found in Supplementary Fig. 2c, e, respectively. In MBC, similar examples are shown for ERα and PR prediction (Supplementary Fig. 2h, j, respectively). The H&E-stained whole slide images (WSI) used for these predictions are shown in Fig. 3g, n for hormone receptor positive FBC and MBC, respectively. Supplementary Fig. 2a, f show the H&E-stained WSI examples for hormone receptor negative FBC and MBC, respectively.

### Tissue architectures with highest attention scores are concordant with receptor expression profiles in both sexes

**Fig. 4:**
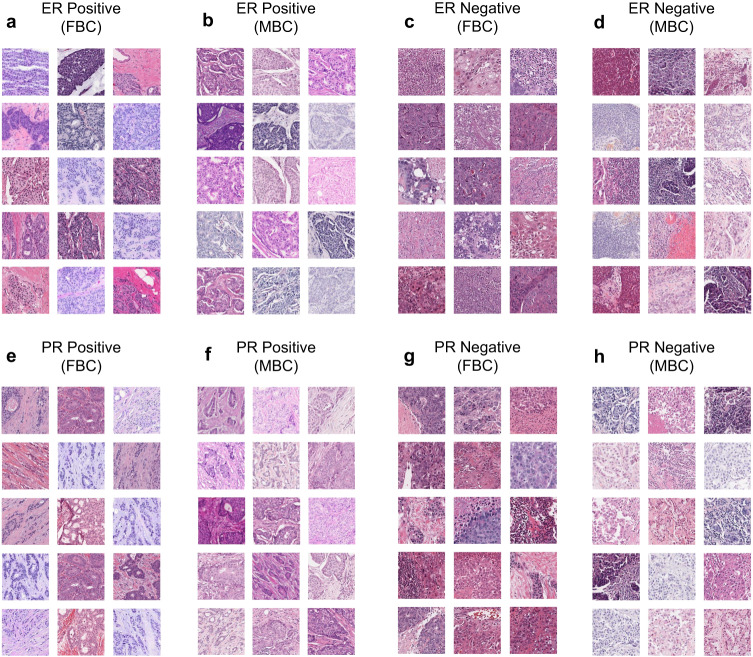
Tiles with top attention scores for ERα and PR prediction in FBC and MBC. Representative examples of tiles with top attention scores in FBC and MBC respectively for the prediction of (**a**, **b**) ERα positivity, (**c**, **d**) ERα negativity, (**e**, **f**) PR positivity, and (**g**, **h**) PR negativity. The area of each tile shown is 256 × 256 μm^2^.

We hypothesized that the histological features associated with ER and PR expression profiles should be similar and investigated whether the prediction models recognised this for both targets. To do this, image tiles with the highest attention scores were identified and collated for each target’s positive and negative classes for FBC internal and external validation cohorts, and the MBC cohort. We observed that the features returning top attention scores for both targets were not only similar but were also conserved for both sexes. Both ERα and PR positive tiles displayed clearly differentiated tumor and stromal regions, while ERα and PR negative tiles showed poorly differentiated cells, high levels of immune infiltration, and necrosis. Collated tiles with top attention scores for both targets in both FBC and MBC cohorts are shown in Fig. 4.

### attMIL-based prediction models are invariant to colour normalisation and do not exhibit domain shift

AUROC values can sometimes misrepresent the performance of a prediction model as they do not provide any information regarding domain shift^33^. To investigate whether either of our prediction models contained domain shift, we visualized the distribution of the model prediction scores for each hormone receptor target in all patient cohorts. Prediction scores for both targets were similarly distributed and free of domain shift for each cohort, regardless of Macenko normalisation. Prediction score distributions for each target in each cohort both with and without normalisation are summarized in Supplementary Figs. 3, 4.

## Discussion

Applications of DL-based techniques in BC pathology have been studied since 2010, including diagnostic (e.g., detection of primary tumor tissue and metastatic deposits, grading, subtyping, assessment of tumor microenvironment etc.), prognostic (e.g., assessment of tumor morphological features with respect to outcome), and predictive (e.g., assessment of therapy response in relation to morphological features) targets/biomarkers^34^. Concerning prediction of hormone receptor status, patch-based^28^, and tissue microarray-based^27^ algorithms have been explored with varying degrees of success. Multiple instance learning (MIL) without an attention component on full-face WSIs has been used to determine ERα status achieving an AUROC of 0.92 – a considerable improvement over the patch-based approach^26^. These techniques could be insightful in understanding the biological behaviour of BC in males and females. However, these have previously been unexplored for that purpose.

We aimed to investigate the generalizability of DL-based techniques in MBC, specifically exploring their applicability across both sexes. Our hypothesis was rooted in the notion that the distinct binding characteristics of ERα and PR could manifest as morphological variances. Consequently, we hypothesized that if there were no substantial variations in morphological features between FBC and MBC, an attMIL model trained on an FBC dataset should perform equally well and exhibit similar accuracy in predicting ERα and PR status in an MBC dataset. Conversely, if there were discernible sex-specific differences in morphological features, predictive models trained on FBC images would likely demonstrate suboptimal performance in an MBC dataset.

We used the attMIL approach with the Retrieval with Clustering-guided Contrastive Learning (RetCCL) based feature extractor to predict ERα and PR in both MBC and FBC. Created on a ResNet50 backbone with a self-supervised learning (SSL) approach, RetCCL uses unlabelled histopathological image data on a large scale to learn universal features that can then be applied to subsequent patch-by-patch WSI retrieval tasks without requiring additional fine tuning. This patch-by-patch retrieval method allows identification of regions-of-interest within each WSI that demonstrate high degrees of similarity with the patches from the query WSI. Consequently, this can generate spatially resolved prediction scores within each WSI allowing visual interpretation of the results^35,36^.

Prediction models were trained on FBC images from the TCGA-BRCA dataset, and their performances were investigated on external FBC and MBC cohorts both with and without Macenko colour normalisation. When applied to the male cohort, performance drops were observed in both models by a large margin irrespective of normalisation status, indicating that ERα and PR status in MBC cannot be predicted with confidence using attMIL models trained on FBC images. In fact, model performances for both ERα and PR prediction in all three cohorts remained invariant to colour normalisation. This disparity in model performances between the sexes supports the growing recognition that male and female BC differs at many levels, including genetic, transcriptomic, and epigenetic^3,16–18,37^, and that these differences may have subtle histopathological manifestations.

In FBC, we showed that our ERα model achieved an AUROC of 0.86 during internal validation and was generalizable to the external FBC cohort. Previous research has suggested that AUROCs approaching 0.9 and exhibiting strong generalizability are highly discriminative^20,38–40^. This standard of performance was achieved by the ERα prediction model in FBC. The prediction model for PR status did not perform to this standard, although PR was predictable during both internal and external validation with statistical significance. This could indicate that either the PR prediction model failed to learn to specifically focus on tumor tissue, or that the tissue architecture surrounding tumor regions could influence making a prediction of PR status. It is worth noting here that for both targets, our attMIL models were free of domain shift in all cohorts, and invariant to Macenko colour normalisation.

Because MBC is rare, we could not accrue sufficiently large numbers of cases from a single centre for this analysis. Hence, we had to obtain these from 7 different sources. Most were ERα-positive, while 7 out of the 198 cases we analysed were negative. This expression pattern is typical in MBC^14^. Unfortunately, this limited our ability to perform AUC by source of image as some of the constituent cohorts were small with <25 cases each and lacked sufficient ground truth, i.e., ERα negativity. However, we qualitatively examined whether the distribution of the prediction scores for both target markers in MBC were similar in each source. The ERα prediction scores for the TCGA and PR prediction scores for BCNTB cases had left-skewed distributions showing that most of the constituent cases were correctly predicted as ERα positive (for TCGA) and PR positive (for BCNTB) with high confidence. It is also important to note that both the BCNTB and TCGA cohorts had small numbers of cases (*n* = 3 and *n* = 12, respectively, after excluding cases with incomplete data). The distribution of prediction scores for both markers in the rest of the cohorts were relatively similar, and we did not observe particularly low performances in any one cohort that could have driven the overall poor performance of the ERα and PR prediction models in the MBC cohort. Obtaining accuracy metrics by source of images when the contributing cohorts are small is challenging, and this has been recognized previously when quantifying site-specific signatures in the TGCA WSI database^41^. Swarm learning may be one way to overcome this in future studies as recently demonstrated^42^, however this has not yet been applied to rare cancers where numbers are limiting.

To ensure the quality and sensitivity of the models towards their respective biomarkers, we conducted a quality control exercise by applying prediction models trained to detect ERα on PR-positive cases, and vice versa, in the external FBC validation cohort. Our approach was grounded on the hypothesis that a reliable biomarker prediction model should exhibit specificity by solely identifying the intended target and not detecting other biomarkers, irrespective of their subcellular localization of expression. In this regard, our results showed exquisite sensitivity; the ERα prediction model had poor power of discrimination in detecting PR status and the reverse was also true. Given that both ERα and PR are classified as nuclear receptors, it is plausible that a predictive model developed for one receptor could potentially identify the other receptor as well. However, our data refuted this, providing further evidence that DL-based techniques are able to detect subtle morphological changes which cannot be distinguished by a human observer.

In both FBC and MBC, ERα and PR positivity is associated with favourable outcomes. ERα and PR negativity, on the other hand, tends to be associated with features of aggressive disease, e.g., poor differentiation, high degree of immune infiltration, and necrosis. We showed that the morphological features that returned the highest attention scores for positive or negative expression of ERα and PR were congruent with the existing pathology. This was true for both sexes. Our algorithm was robust against artefacts (e.g., folding, tearing, pathologists’ ink) in the WSIs, returning low attention scores for both ERα and PR prediction. However, we sporadically observed high attention scores being returned for morphological features external to the breast tissue, such as the skin edge. In addition, while ERα expression is typically dichotomised as a binary variable, updated guidelines from the American Society of Clinical Oncology and College of American Pathologists (ASCO-CAP) propose that breast tumours with low levels of ERα expression (1–10%) be reported as ER-low-positive^43^. Indeed, recent data has demonstrated that these tumours behave like ERα-negative breast cancer and are a clinically and biologically distinct subgroup^44^. This requires consideration in future studies.

We acknowledge that our study was limited by the lack of an MBC validation cohort. A further limitation of our study was not evaluating HER2 (human epidermal growth factor receptor 2), which is part of the clinical management workflow in BC. HER2 expression is quantified primarily by IHC with scores of 0/1+ (negative), 2+ (equivocal) and 3+ (positive). Cases with equivocal expression need to undergo fluorescent/bright-field in-situ hybridization assays (ISH) to confirm gene amplification, which then ultimately classifies these cases as positive or negative^45^. While an important biomarker in BC, HER2 poses a challenge for DL-based predictions directly from H&E-based images. Its expression is seen in around 15% of women^45^, and is especially rare in males (0–9%)^1^. Furthermore, most FBC clinical datasets with HER2 data include equivocal cases that lack confirmatory ISH testing. Therefore, they introduce a degree of ambiguity in the ground truth. This is exacerbated in MBC due to the small number of cases that express HER2. Taking these challenges into account, testing the predictability of HER2 status in BC of either sex using DL-based techniques would require improved curation of datasets, large multi-centric cohorts, and multimodal approaches which takes both proteomic and genetic data into account.

To conclude, we showed that attMIL workflows have the potential to predict ERα status in FBC with accuracy levels that are clinically relevant, and that spatial resolution of attention scores is concordant with IHC staining patterns of both ERα and PR. However, attMIL-based prediction models trained on FBC images were ineffective when applied to MBC datasets. These results align with the growing recognition that sex can differentially influence the behaviour of cancers in general, and breast cancer in particular^46,47^. Our findings support previous evidence that male and female BC are different on many levels, and suggest that subtleties in BC tissue architecture that are invisible to the human eye but detectable by DL may also be sex specific.

## Methods

### Ethical approval and consent to participate

This study is a retrospective analysis of digital images of anonymized archival tissue samples. The experiments in this study were carried out according to the Declaration of Helsinki and the International Ethical Guidelines for Biomedical Research Involving Human Subjects by the Council for International Organizations of Medical Sciences (CIOMS). The Ethics Board at the Medical Faculty of the Technical University of Dresden approved of the overall analysis in this study. The patient sample collection in each cohort was separately approved by the respective institutional ethics boards as follows: the Leeds (West) Research Ethics Committee (06/Q125/156), NHS Grampian Tissue Bank Committee (TR000292), Greater Glasgow Health Board (TR000269), Northern Ireland Biobank (NIB22-0007), Wales Cancer Biobank (22-005), and Breast Cancer Now Tissue Bank Access Committee (TR249). All patients provided written informed consent.

Two cohorts of FBC patients were used: a training set from The Cancer Genome Atlas – Breast Cancer (TCGA-BRCA) dataset (*n* = 1085), followed by a combined validation set of FBC cases (*n* = 192) compiled from: Breast Cancer Now Tissue Bank (*n* = 58) and the Clinical Proteomic Tumor Analysis Consortium – Breast Cancer (CPTAC-BRCA) dataset (*n* = 134). For MBC, 6 cohorts were used, totalling 245 cases from: the Male Breast Cancer Consortium (MBCC; *n* = 126), NHS Greater Glasgow and Clyde (NHSGGC) Biorepository (*n* = 40), NHS Grampian (NHSG) Biorepository (*n* = 21), Northern Ireland Biobank (NIB; *n* = 25)^48^, Wales Cancer Biobank (WCB; *n* = 10)^49^, Breast Cancer Now Tissue Bank (BCNTB; *n* = 11), and TCGA-BRCA dataset (*n* = 12). The initial combined cohort of 245 MBC cases were manually screened for ERα and PR status. Only cases with known ERα and PR status were included (*n* = 198). MBC cases were scored using the Allred method^50^, with scores ≥3 considered positive. FBC scores came from a range of sources in the form of binary values that did not have defined cut-offs for all cases.

### Image preprocessing

All H&E-stained WSIs used in our analyses were pre-processed following the “Aachen protocol for deep learning histopathology”^51^. All WSIs underwent tessellation into tiles with edge lengths of 256 μm, and pixel area of 224 px * 224 px with an effective resolution of 1.14 μm/px. Blurry tiles and tiles containing background were removed automatically using the canny edge detection technique within the OpenCV package in Python^52^. These tiles were then colour-normalised following the Macenko method to remove any bias arising from differences in staining between cohorts^53^. We did not apply any manual annotations and our analysis was not restricted to the tumor region alone. All models were trained solely on the basis of slide-level target labels. Subsequent steps of feature extraction, model training, and deployment were performed on both colour-normalised and unnormalised tiles.

### Experimental setup

Attention-based multiple instance learning (attMIL)^54,55^ models were used to predict ERα and PR binary classification status in both FBC and MBC patient samples.

Models were trained on FBC H&E-stained WSIs from the TCGA-BRCA cohort (*n* = 1085) using biomarker-stratified five-fold cross-validation. A quarter of the patients in each training fold were reserved as a validation dataset to monitor overfitting during the training process. Trained models were externally validated on two cohorts: the external FBC validation cohort (*n* = 192) and the MBC cohort (*n* = 198).

### Feature extraction and implementation of attMIL

Feature vectors for images within the attMIL procedure were extracted using RetCCL, an SSL-based feature extractor with a ResNet50 backbone pretrained on a large histopathology dataset (https://github.com/Xiyue-Wang/RetCCL)^35,54^. During training, model parameters were updated using the Adam optimizer^56^ with 1% weight decay. Momenta and learning rates were scheduled using the “fit one cycle” procedure over a total of 32 epochs as made available in fastai (https://docs.fast.ai/callback.schedule.html)^57,58^. The maximal learning rate was 1e-4. Over the first eight epochs, the learning rate sinusoidally increased from 1/25 of the maximum to the maximum and sinusoidally decreased to 1e-6 of the maximum over the remaining epochs. With the same modulation, the optimizer’s momentum was increased from 0.85 to 0.95 and returned to 0.85. The batch size used for updating model weights incrementally was 64 patients.

To implement attMIL, a fully connected layer followed by a Rectified Linear Unit (ReLU) were used to embed feature vectors in a 256-dimensional space. Then, these embedded vectors were passed through a linear layer to output a further 256-dimensional feature vector (*h*_*k*_), where *k* is the index of each tile. The attention score (*a*_*k*_) for the *k*-th tile was calculated as:

1 $${a}_{k}=\frac{\exp \{{w}^{T}\,\tanh (V{h}_{k})\}}{{\sum }_{j=1}^{K}\exp \{{w}^{T}\,\tanh (V{h}_{j})\}}$$

where *h* ∈ *R*^*256*^, *V* ∈ *R*^*128×256*^, *w* ∈ *R*^*128*^, and *K* is the maximum number of tiles resampled per epoch per patient. We used *K* = 512 tiles per patient. Then, MIL pooling operation was applied as follows:

2 $${h}_{{sum}}={\sum }_{i=1}^{K}{a}_{i}{h}_{i}$$

where *h*_*i*_ is the *i*-th tile’s embedding. The final prediction score for each patient was obtained by passing each batch of *h*_*sum*_ values through a BatchNorm1D layer first, and then a Dropout layer with *p* = 50%. Then, *h*_*sum*_ values were passed through a fully connected layer with 2-dimensional output, followed by a softmax layer to obtain the final prediction scores.

**Fig. 5:**
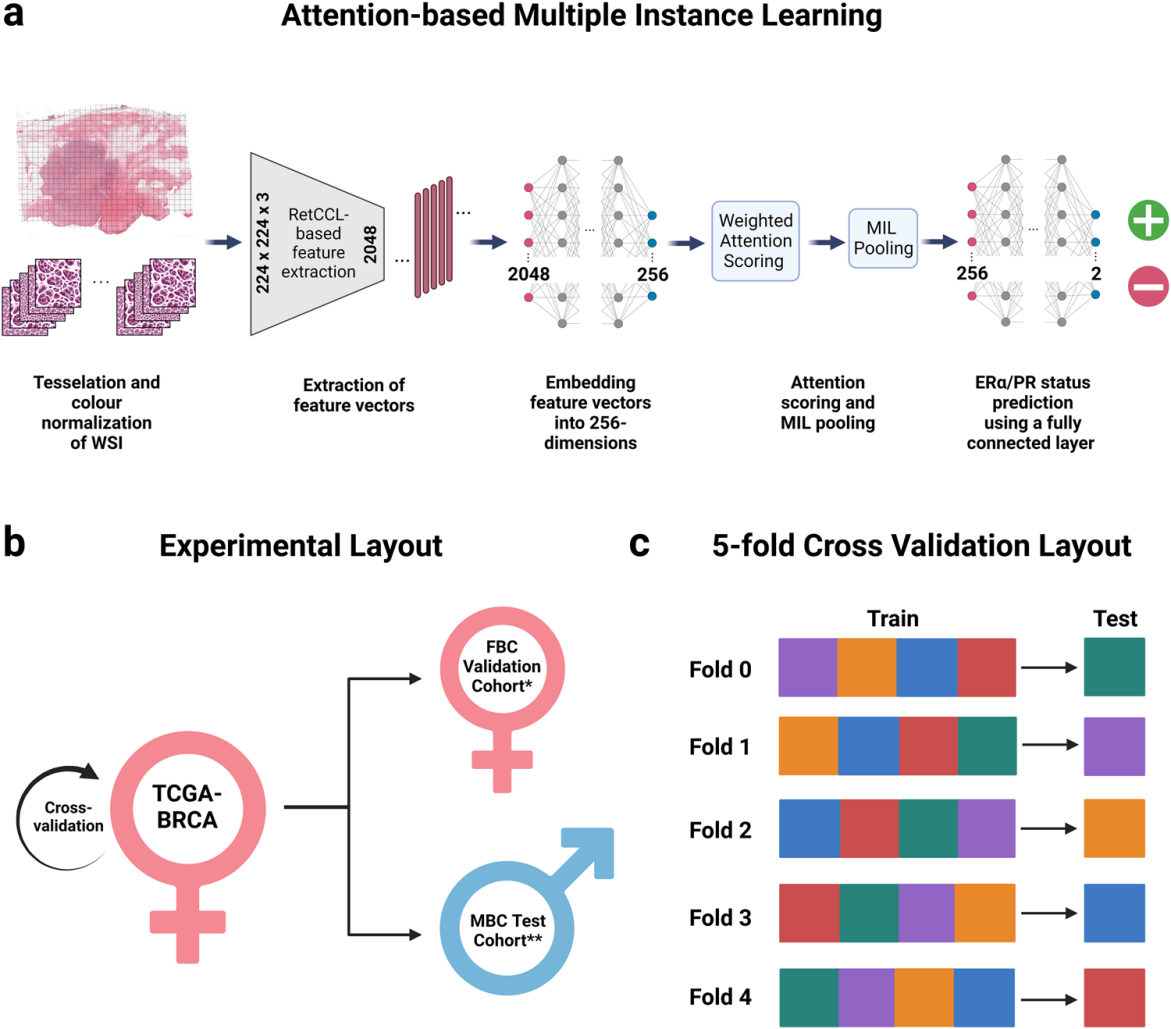
Experimental pipeline of attMIL-based prediction models. Schematic of experimental setup employed in this study showing the (**a**) architecture of the attention-based multiple instance learning pipeline; (**b**) cohorts used for training and cross-validation (TCGA-BRCA FBC), and external validation (FBC and MBC); (**c**) schematic of 5-fold cross-validation during which the cohort is divided into 5 equal sets. In each fold, the model is trained on 4/5th of the data and tested on the remaining 1/5th. This is repeated 5 times, such that each set is used as the test set once. This ensures that the model is tested on multiple and mutually exclusive subsets of the data, providing a representative evaluation of the dataset. Figure created with BioRender.com. *FBC external validation cohort composed of cases from Breast Cancer Now Tissue Bank and CPTAC-BRCA dataset. **MBC composed of cases from the Male Breast Cancer Consortium, NHS Greater Glasgow and Clyde Biorepository, NHS Grampian Biorepository, Northern Ireland Biobank, Wales Cancer Biobank, Breast Cancer Now Tissue Bank, and TCGA-BRCA dataset.

The full experimental strategy is outlined in Fig. 5.

### Explainability and biological validation with immunohistochemistry

For easy visualization of our prediction models, we generated spatially resolved heatmaps showing the distribution of attention and classification scores for each tile within each WSI, for each target. Feature vectors for 32 × 32-pixel fields were extracted from the WSI using the RetCCL algorithm^35^. Attention and classification scores were calculated for each image region, and normalised within each patient cohort. Based on the resulting scores, attention and prediction score heatmaps for each patient were generated. For the former, a purple (low) to gold (high) colour scale was used to visualise the spatial distribution of the attention scores in a WSI. For the latter, a blue to red colour scale was used, with blue indicating negative classification and red indicating positive classification. Each heatmap was overlaid on its corresponding H&E WSI, allowing visual interpretation of underlying morphological features, correlating with classification types and high attention scores. We also matched classification heatmaps to immunohistochemically stained sections for ERα and PR from these cases.

### Statistics

The primary statistical endpoint for our analyses was the AUROC determined at patient-level. Since we only performed binary classification, AUROCs were identical for both “positive” and “negative” classes for each target. Therefore, we only reported AUROCs for “positive” classes within each target. Distribution of patient level prediction scores for each target was further visualized using density plots, which were also used to quantify domain shift between models trained and tested on normalised *vs*. unnormalised tiles. All statistical tasks were performed using Python 3.11 and R 4.3.0.

### Reporting summary

Further information on research design is available in the Nature Research Reporting Summary linked to this article.

### Supplementary information

Supplementary Files

Reporting Summary

## Data Availability

All images included in the training set (*n* = 1085) are available at https://portal.gdc.cancer.gov/ and information about their hormone receptor status is available at https://www.cbioportal.org/. Part of the female breast cancer external validation set (*n* = 134) images and their associated clinical information are available at https://www.cancerimagingarchive.net/collections/. All other data are available from the principal investigators upon reasonable request.

## Code availability

All code used in this article is open source and available at https://github.com/KatherLab/marugoto.
