# Supplementary material for: Prediction models for hormone receptor status in female breast cancer do not extend to males: further evidence of sex-based disparity in breast cancer
Source: NPJ Breast Cancer. 2023 Nov 8;9:91. doi: 10.1038/s41523-023-00599-y (PMC10632426; doi:10.1038/s41523-023-00599-y)
Supplement: Supplementary file 1 — Supplementary Files [file 41523_2023_599_MOESM1_ESM.docx]

# Supplementary Figures


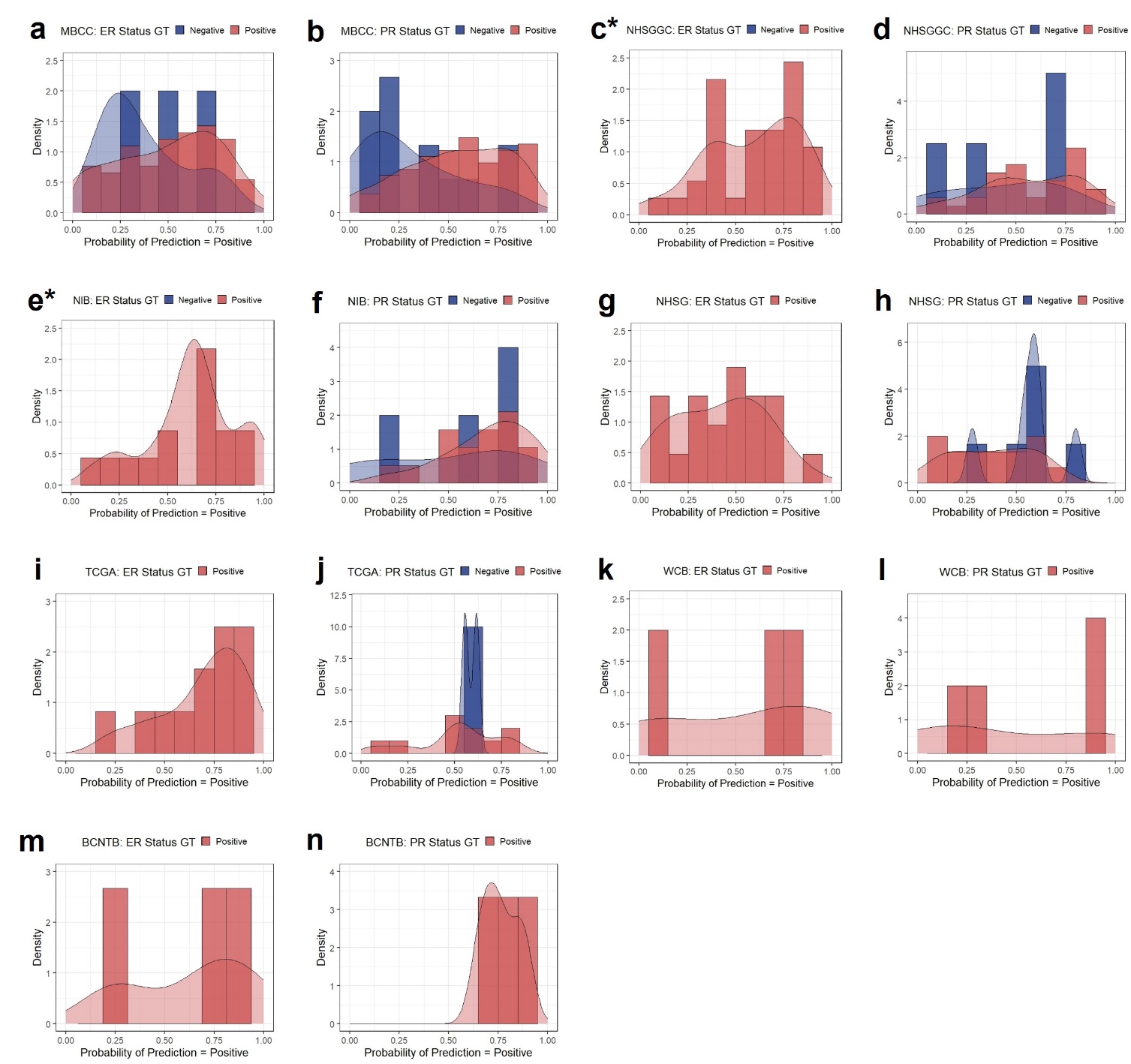


## Supplementary Figure 1: Visualization of the distribution of prediction scores in MBC by source of images

Distribution of prediction scores for ERα and PR models, respectively, for MBC cases originating from **(a-b)** MBCC, **(c-d)** NHSGGC, **(e-f)** NIB, **(g-h)** NHSG, **(i-j)** TCGA, **(k-l)** WCB, and **(m-n)** BCNTB.

*the NHSGGC and NIB cohorts had 1 ERα negative case each which have not been visualized in the density plots. This is because creation of density curves requires a minimum of 3 data points.


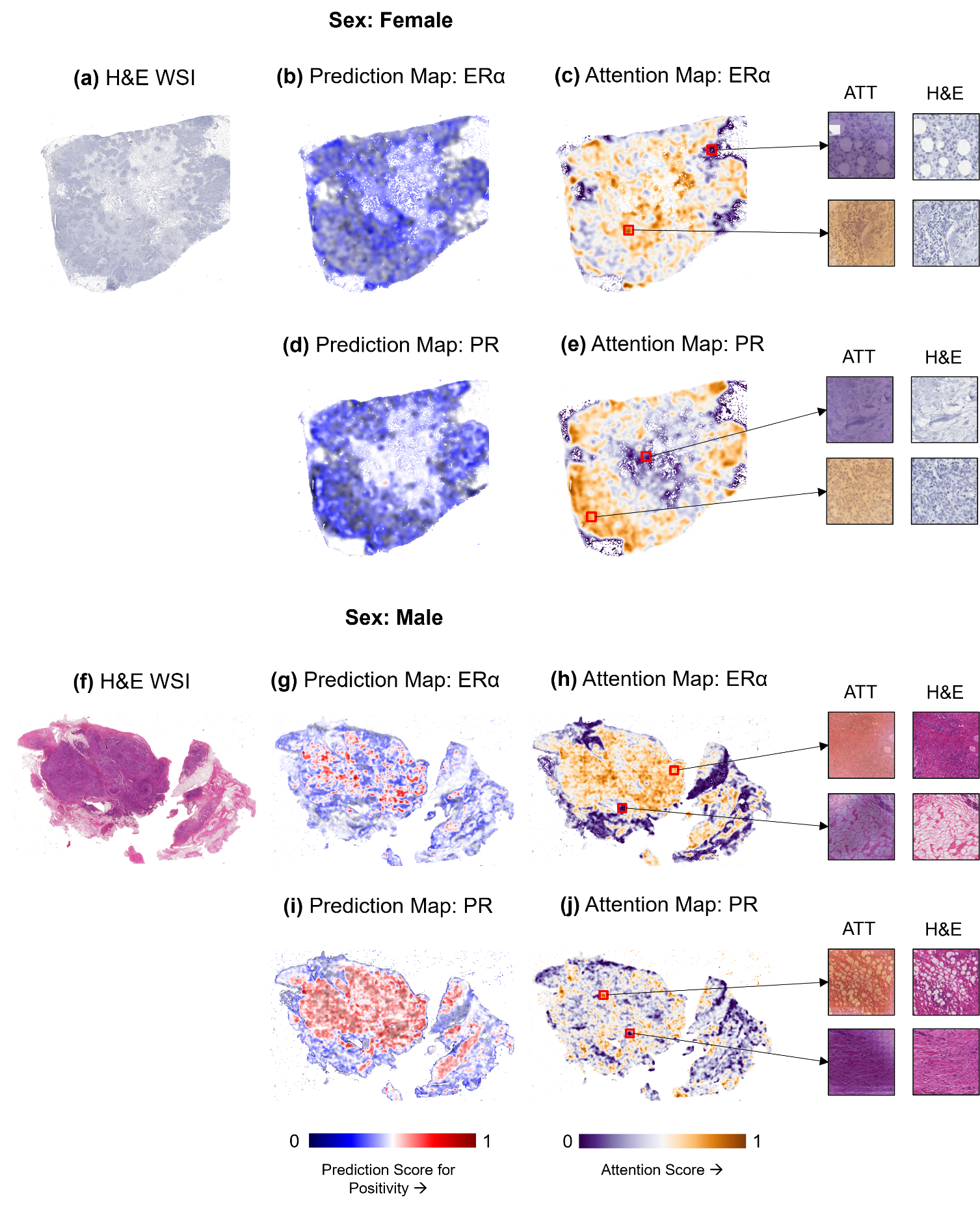


## Supplementary Figure 2: Heatmaps showing spatial resolution of attention and prediction scores in ERα and PR negative FBC and MBC WSIs and the H&E WSIs from which these heatmaps were generated

Heatmaps showing spatial resolution of attention (ATT) and prediction scores for FBC **(top)** and MBC **(bottom)** prediction models with respective adjacent views of **(a, f)** H&E-stained WSI, **(b, g)** prediction score map for ERα, **(c, h)** attention score map for ERα, **(d, i)** prediction score map for PR, and **(e, j)** attention score map for PR with magnified view of representative tiles for high and low attention regions. The attention map showcases the relevant morphological features with high attention regions in gold and low attention regions in purple, irrespective of the final prediction. The prediction map highlights the relevance of each tile in making a prediction of the target receptor status with positivity represented in red, and negativity in blue. The statuses of both target receptors were predicted correctly in the FBC WSI. In the MBC WSI, the overall ERα status was predicted correctly although certain areas within the WSI were predicted to be positive. The PR status was incorrectly predicted with the entire tumor area receiving a positive prediction.


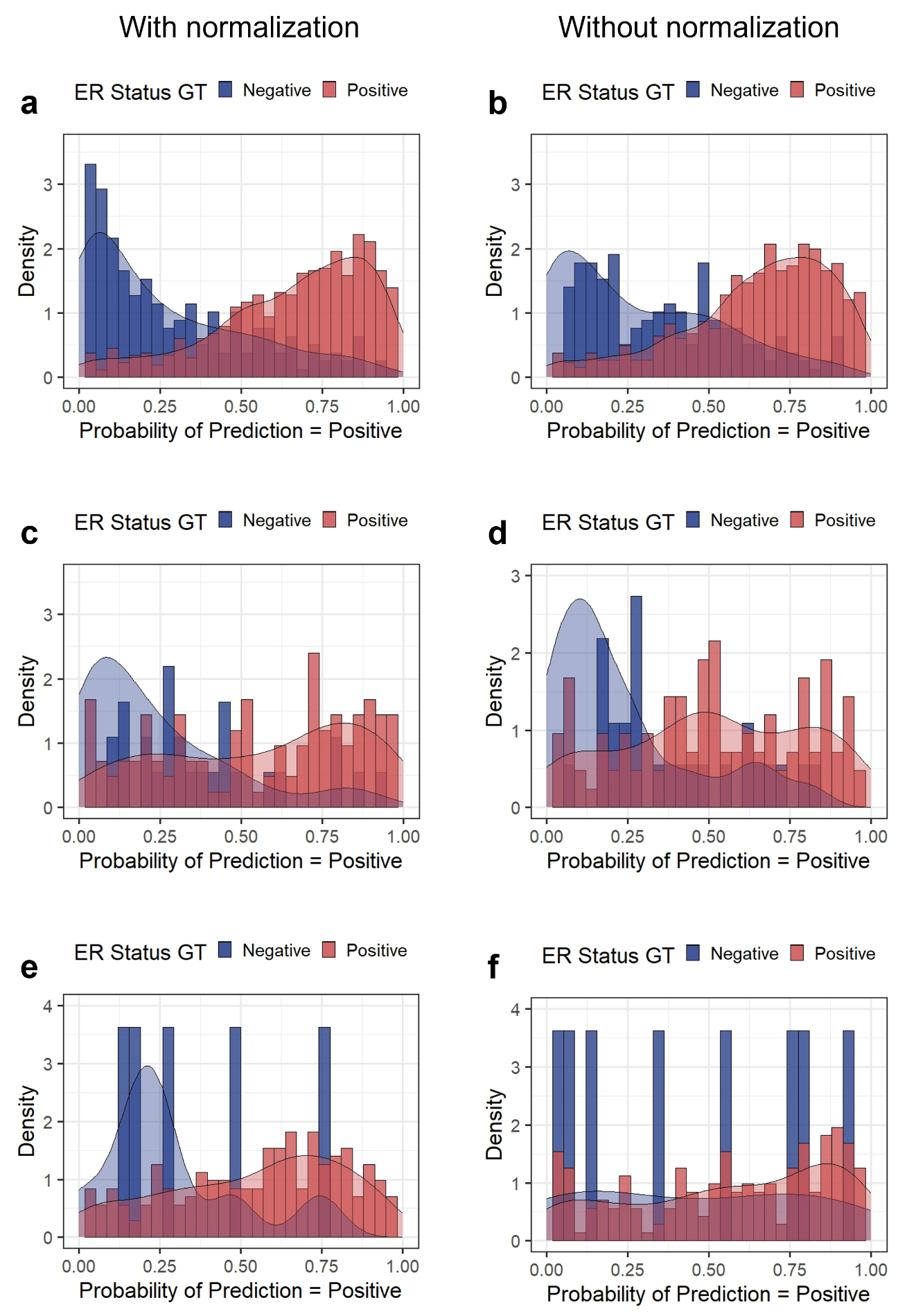


## Supplementary Figure 3: Visualization of domain shift between ERα prediction models with and without Macenko normalisation

Distribution of ERα model prediction scores with **(left)** and without **(right)** Macenko normalisation for **(a-b)** FBC TCGA internal validation cohort, **(c-d)** FBC external validation cohort, and **(e-f)** MBC cohort. GT = Ground Truth

##


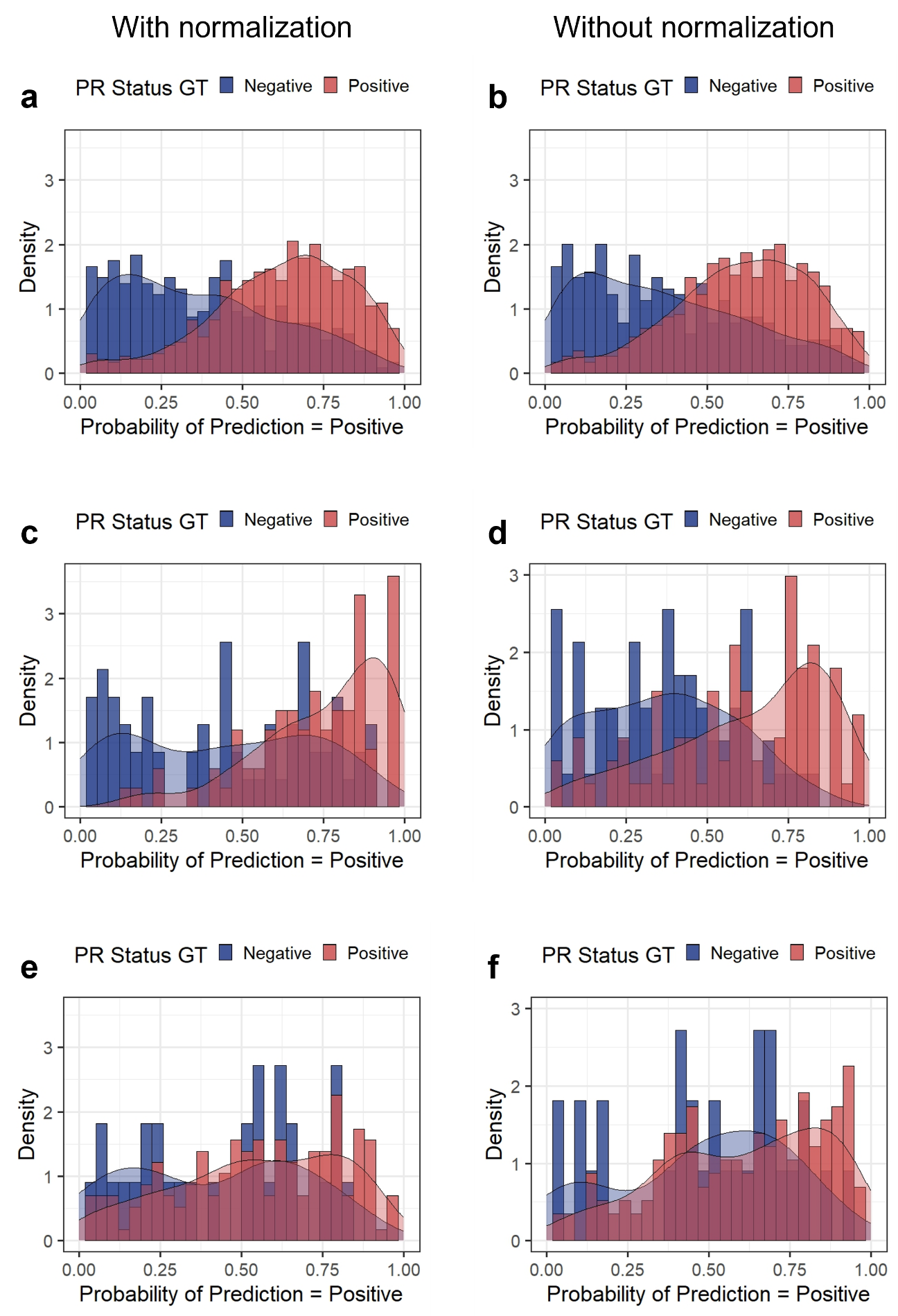


## Supplementary Figure 4: Visualization of domain shift between the PR prediction models with and without Macenko normalisation

Distribution of PR model prediction scores with **(left)** and without **(right)** Macenko normalisation for **(a-b)** FBC TCGA internal validation cohort, **(c-d)** FBC external validation cohort, and **(e-f)** MBC cohort. GT = Ground Truth

# Supplementary Tables

## Supplementary Table 1: Performance metrics of prediction models for ERα and PR in each female and male breast cancer cohort

| **Target** | **Training Set** | **Test Set** | **AUROC** | **AUPRC (+ve)** | **AUPRC (-ve)** | **Normalisation** |
| --- | --- | --- | --- | --- | --- | --- |
| ERα | FBC TCGA | FBC TCGA | 0.86 ± 0.02 | 0.95 ± 0.01 | 0.67 ± 0.08 | Macenko |
|  |  |  | 0.86 ± 0.05 | 0.94 ± 0.02 | 0.67 ± 0.11 | None |
| ERα | FBC TCGA | FBC EXT-VAL | 0.78 ± 0.03 | 0.88 ± 0.03 | 0.58 ± 0.04 | Macenko |
|  |  |  | 0.78 ± 0.05 | 0.89 ± 0.03 | 0.59 ± 0.07 | None |
| ERα | FBC TCGA | MBC | 0.66 ± 0.14 | 0.98 ± 0.01 | 0.11 ± 0.04 | Macenko |
|  |  |  | 0.69 ± 0.09 | 0.98 ± 0.01 | 0.14 ± 0.03 | None |
| PR | FBC TCGA | FBC TCGA | 0.76 ± 0.03 | 0.83 ± 0.03 | 0.66 ± 0.05 | Macenko |
|  |  |  | 0.78 ± 0.02 | 0.85 ± 0.03 | 0.67 ± 0.05 | None |
| PR | FBC TCGA | FBC EXT-VAL | 0.80 ± 0.04 | 0.86 ± 0.04 | 0.74 ± 0.05 | Macenko |
|  |  |  | 0.76 ± 0.03 | 0.79 ± 0.04 | 0.71 ± 0.02 | None |
| PR | FBC TCGA | MBC | 0.63 ± 0.04 | 0.89 ± 0.02 | 0.27 ± 0.02 | Macenko |
|  |  |  | 0.62 ± 0.03 | 0.90 ± 0.01 | 0.28 ± 0.02 | None |

*Abbreviations: ERα = Estrogen Receptor α; PR = Progesterone Receptor; FBC = Female Breast Cancer; MBC = Male Breast Cancer; TCGA = The Cancer Genome Atlas; EXT-VAL = External Validation; AUROC = Area Under Receiver Operating Curve; AUPRC = Area Under Precision Recall Curve*
