# Supplementary material for: Prediction models for hormone receptor status in female breast cancer do not extend to males: further evidence of sex-based disparity in breast cancer
Source: NPJ Breast Cancer. 2023 Nov 8;9:91. doi: 10.1038/s41523-023-00599-y (PMC10632426; doi:10.1038/s41523-023-00599-y)
Supplement: Supplementary file 2 — Reporting Summary [file 41523_2023_599_MOESM2_ESM.pdf]

nature portfolio

Corresponding author(s): Professor Dr. med. Jakob Nikolas Kather
Professor Valerie Speirs

Last updated by author(s): Jul 31, 2023

Reporting Summary

Nature Portfolio wishes to improve the reproducibility of the work that we publish. This form provides structure for consistency and transparency in reporting. For further information on Nature Portfolio policies, see our Editorial Policies and the Editorial Policy Checklist.

Statistics

For all statistical analyses, confirm that the following items are present in the figure legend, table legend, main text, or Methods section.

n/a	Confirmed	
☐	☒	The exact sample size (*n*) for each experimental group/condition, given as a discrete number and unit of measurement
☐	☒	A statement on whether measurements were taken from distinct samples or whether the same sample was measured repeatedly
☒	☐	The statistical test(s) used AND whether they are one- or two-sided<br>*Only common tests should be described solely by name; describe more complex techniques in the Methods section.*
☐	☒	A description of all covariates tested
☒	☐	A description of any assumptions or corrections, such as tests of normality and adjustment for multiple comparisons
☒	☐	A full description of the statistical parameters including central tendency (e.g. means) or other basic estimates (e.g. regression coefficient) AND variation (e.g. standard deviation) or associated estimates of uncertainty (e.g. confidence intervals)
☒	☐	For null hypothesis testing, the test statistic (e.g. *F*, *t*, *r*) with confidence intervals, effect sizes, degrees of freedom and *P* value noted<br>*Give P values as exact values whenever suitable.*
☒	☐	For Bayesian analysis, information on the choice of priors and Markov chain Monte Carlo settings
☒	☐	For hierarchical and complex designs, identification of the appropriate level for tests and full reporting of outcomes
☒	☐	Estimates of effect sizes (e.g. Cohen's *d*, Pearson's *r*), indicating how they were calculated

Our web collection on statistics for biologists contains articles on many of the points above.

Software and code

Policy information about availability of computer code

Data collection	No software was used.
Data analysis	All code used in this article is open source and available at https://github.com/KatherLab/marugoto. All numerical analysis were performed using Python 3.11 and R 4.3.0.

For manuscripts utilizing custom algorithms or software that are central to the research but not yet described in published literature, software must be made available to editors and reviewers. We strongly encourage code deposition in a community repository (e.g. GitHub). See the Nature Portfolio guidelines for submitting code & software for further information.

Data

Policy information about availability of data

All manuscripts must include a data availability statement. This statement should provide the following information, where applicable:

- Accession codes, unique identifiers, or web links for publicly available datasets
- A description of any restrictions on data availability
- For clinical datasets or third party data, please ensure that the statement adheres to our policy

All images included in the training set (n = 1085) are available at (https://portal.gdc.cancer.gov/) and information about their hormone receptor status is available at (https://www.cbioportal.org/). Part of the female breast cancer external validation set (n = 134) images and their associated clinical information are available at (https://www.cancerimagingarchive.net/collections/). All other data are available from the principal investigators upon reasonable request.

Research involving human participants, their data, or biological material

Policy information about studies with human participants or human data. See also policy information about sex, gender (identity/presentation), and sexual orientation and race, ethnicity and racism.

Reporting on sex and gender	The main hypothesis of this study was that sex-based differences in breast cancer could have morphological manifestations that are undetectable to human observers but could be computationally resolved. Therefore, our study was primarily predicated on the pathological differences in breast cancer driven by the biological attribute of sex. We confirm that we have only used the term "sex" and not the socially defined identity of "gender".<br>These data were collected either from publicly available repositories or in the form of pseudonymised data from various centres. In the latter case, each centre conducted and approved their own ethical review (detailed in the Methods section of the manuscript).
Reporting on race, ethnicity, or other socially relevant groupings	As described above, the only variable used for group allocation was sex, as the major focus of our investigation was driven by this variable. No other groupings were used in this study.
Population characteristics	See above
Recruitment	All data used in this study were collected either from publicly available repositories or in the form of pseudonymised data from various biobanks. In the latter case, each biobank conducted and approved their own ethical review (detailed in the Methods section of the manuscript).
Ethics oversight	The Ethics Board at the Medical Faculty of the Technical University of Dresden approved of the overall analysis in this study. The patient sample collection in each cohort was separately approved by the respective institutional ethics boards as follows: the Leeds (West) Research Ethics Committee (06/Q125/156), NHS Grampian Tissue Bank Committee (TR000292), Greater Glasgow Health Board (TR000269), Northern Ireland Biobank (NIB22-0007), Wales Cancer Biobank (22-005), and Breast Cancer Now Tissue Bank Access Committee (TR249).

Note that full information on the approval of the study protocol must also be provided in the manuscript.

Field-specific reporting

Please select the one below that is the best fit for your research. If you are not sure, read the appropriate sections before making your selection.

☒ Life sciences ☐ Behavioural & social sciences ☐ Ecological, evolutionary & environmental sciences

For a reference copy of the document with all sections, see nature.com/documents/nr-reporting-summary-flat.pdf

Life sciences study design

All studies must disclose on these points even when the disclosure is negative.

Sample size	Our study focused on male breast cancer which is a rare disease. As is typical of rare cancers, our sample size was driven by the numbers of cases available in the seven centres from which we accrued the cases for our study.
Data exclusions	Our study was dependent on the presence of estrogen and progesterone receptor status data. Cases that did not have these data were excluded from the study.
Replication	The male breast cancer cohort in our study was accrued in stages, which led to these experiments being conducted each time new data became available. There were no changes in the patient level prediction metrics in the male breast cancer cases that were common to each experimental replicate. In addition, the female breast cancer cohort did not change in any of these experimental replicates, and returned the same accuracy metrics every time. Furthermore, the sex-based disparities in model performances were consistently observed every time the experiments were conducted. Finally, the same experiments were conducted initially at the Technical University of Dresden using a system with 128GB RAM and two NVIDIA Quadro RTX 6000 graphics processing units (GPUs), and later at the University of Aberdeen using a system with 64GB RAM and one NVIDIA GeForce RTX 2070 GPU, without any change in results.
Randomization	Randomization was not suitable as the main hypothesis of this study is driven by the sex of the included cases. Therefore, all cases in this study were allocated into experimental groups solely based on their sex (male or female).
Blinding	Blinding during group allocation was not appropriate in our study. As described above, since our study is focused on sex-based differences in breast cancer, cases were grouped by their sex alone. However, the prediction models used in this study were blinded to the ground truth data during analysis. This data was only used during the generation of receiver operating characteristic and precision recall curves.

Reporting for specific materials, systems and methods

We require information from authors about some types of materials, experimental systems and methods used in many studies. Here, indicate whether each material, system or method listed is relevant to your study. If you are not sure if a list item applies to your research, read the appropriate section before selecting a response.

Materials & experimental systems

n/a	Involved in the study
☒	☐ Antibodies
☒	☐ Eukaryotic cell lines
☒	☐ Palaeontology and archaeology
☒	☐ Animals and other organisms
☒	☐ Clinical data
☒	☐ Dual use research of concern
☒	☐ Plants

Methods

n/a	Involved in the study
☒	☐ ChIP-seq
☒	☐ Flow cytometry
☒	☐ MRI-based neuroimaging
